# Size-frequency distribution of coral assemblages in insular shallow reefs of the Mexican Caribbean using underwater photogrammetry

**DOI:** 10.7717/peerj.8957

**Published:** 2020-04-17

**Authors:** Roberto C. Hernández-Landa, Erick Barrera-Falcon, Rodolfo Rioja-Nieto

**Affiliations:** 1 Laboratorio de Análisis Espacial de Zonas Costeras (COSTALAB), Unidad Multidisciplinaria de Docencia e Investigación-Sisal, Facultad de Ciencias, Universidad Nacional Autónoma de México, Sierra Papacal, Yucatán, México; 2 Posgrado en Ciencias del Mar y Limnología, Universidad Nacional Autónoma de México, Mérida, Yucatán, México; 3 Escuela Nacional de Estudios Superiores, Unidad Mérida, Universidad Nacional Autónoma de México, Mérida, Yucatán, Mexico

**Keywords:** Digital photogrammetry, Coral reefs, Spatial analysis, Minimum sampling area, Size-frequency distribution, Shallow reefs

## Abstract

The characterisation of changes in coral communities depends heavily on systematic monitoring programs and the collection of necessary metrics to assess reef health. Coral cover is the most used metric to determine reef health. The current organizational shift in coral requires the evaluation of complementary metrics, such as colony size and frequency distributions, which help to infer the responses of the coral populations to local stress or larger scale environmental changes. In this study, underwater digital photogrammetry techniques were used to assess the live cover of all coral colonies ≥3 cm^2^ and determine the size-frequency distribution of the dominant species in the shallow reefs of the Cozumel Reefs National Park (CRNP). In addition, the minimum sampling area (m^2^) needed to obtain a representative sample of the local species pool was estimated. Areas between 550 and 825 m^2^ per reef were photographed to generate high-resolution digital ortho-mosaics. The live area of the colonies was digitised to generate community matrices of species and abundance. EstimateS software was used to generate accumulation curves and diversity (Shannon *H*′) at increasing area intervals. Chi-Square tests (*χ*^2^, *p* = 0.05) were used to compare the observed *vs* estimated species richness. Spearman’s coefficients (*r*_*s*_), were calculated to correlate the increase in sampling area (m^2^) *vs H*′, and the Clench’s function was used to validate the observed richness (*R*^2^ = 1 and *R* > 90%). SIMPER analysis was performed to identify dominant species. Comparisons in terms of abundance, coral cover and size-frequencies were performed with Kruskal-Wallis (*H* test, *p* = 0.05), and paired Mann-Whitney (*U* test, *p* = 0.05). In order to obtain 90% of the species richness, a minimum sampling area of 374 m^2^is needed. This sampling area could be used in shallow Caribbean reefs with similar characteristics. Twelve (mainly non-massive) species: *Agaricia agaricites, A humilis, A. tenuifolia, Eusmilia fastigiata, Meandrina meandrites, Montastrea cavernosa, Orbicella annularis, Porites astreoides, P. porites, Pseudodiploria strigosa, Siderastrea radians* and*S. siderea*, were dominant in terms of abundance and coral cover. A significant increase (*p* < 0.05) in the number of colonies and live coral (m^2^) was observed from north to south of the study area. Furthermore, a wide intraspecific variation of size-frequency, even between adjacent reefs, was also observed. The size-frequency distributions presented positive skewness and negative kurtosis, which are related to stable populations, with a greater number of young colonies and a constant input of recruits. Considering the increase in disturbances in the Caribbean and the appearance of a new coral disease, digital photogrammetry techniques allow coral community characteristics to be assessed at high spatial resolutions and over large scales, which would be complementary to conventional monitoring programs.

## Introduction

Coral reefs are among the most ecologically and economically productive marine ecosystems. However, over the last four decades the condition of reefs around the world has declined severely (Raeaka-Kudla, 1997; Mumby & Harborne, 2010). Coral reefs are locally and regionally subjected to a wide range of natural and human stressors, including overfishing, coastal development, algal blooms, disease outbreaks, invasive species, hurricanes and the effects of climate change, which have generated dramatic shifts in the community structure and composition (Glynn, 1984; Hoegh-Guldberg, 1999; Gardner et al., 2003; Bellwood et al., 2004; Carpenter et al., 2008; Obura & Grimsdith, 2009). The environmental goods and services obtained from coral reef ecosystems (e.g., coastal protection against hurricanes, food and income for local communities) are seriously threatened (Moberg & Folke, 1999; Hoegh-Guldberg et al., 2007; Franklin et al., 2018). The synergy of multiple stress factors has driven a phase shift that has altered the balance between corals and algae, which is characterised by the loss of coral cover and a substantial increase in macroalgae (Jackson et al., 2014). In addition, other shifts in the hierarchical dominance of coral species have occurred in the Caribbean. The previously dominant reef-builders, such as the branching species, *Acropora cervicornis* and *A. palmata*, or massive corals such as the *Orbicella* complex, have been disproportionately susceptible to several disturbances (e.g., bleaching events, disease outbreaks, among others) (Bruckner & Bruckner, 2006; Weil, Croquer & Urreiztieta, 2009), and opportunistic, small-sized coral species, such as *Agaricia agaricites* and *Porites astreoides*, have increased in abundance, dominating the current coral assemblages throughout the Caribbean (Alvarez-Filip et al., 2013; Estrada-Saldívar et al., 2019).

Recently, new stress factors have appeared in the Caribbean region, including the atypical massive arrival of *Sargassum spp*, observed over the last few years (Rioja-Nieto & Álvarez Filip, 2018; Cabanillas-Terán et al., 2019), and the emergence of a new coral disease observed for the first time in Florida’s coral reefs (Florida Department of Environmental Protection, 2019), locally known as “White syndrome” in the Mesoamerican Reef System (MAR) (Sociedad Mexicana de Arrecifes Coralinos, 2015). White syndrome has expanded rapidly, affecting a wide number of scleractinian corals species, among them important reef-building species (e.g., *Orbicella sp, Colpophyllia natans, Pseudodiploria sp*, among others). The rapid expansion of this disease threatens the marine biodiversity and socio-economic activities of the region (Precht et al., 2016; Van Woesik & Randall, 2017; Healthy reefs, 2019) and could lead to new shifts in the condition and structure of coral assemblages. Conservation strategies are critical to maintain the health and environmental services of coral reefs. These strategies depend heavily on the different methods employed to obtain metrics which are used to assess the condition of the coral reef communities (Hughes et al., 2010; Turner et al., 2015), and are collected as part of monitoring programs and local action plans.

Most monitoring programs have focused on the trajectory of coral cover loss (Jackson, 1997; Gardner et al., 2003), on the partial or total mortality of key coral species (Aronson & Precht, 2001), or on the coral-macroalgae balance (Knowlton, 2001; Kramer, 2003). In the majority of these studies, coral cover is the most used metric to assess coral communities and determine the health of a reef (Gardner et al., 2003; Hodgson et al., 2006; Hill & Wilkinson, 2004). However, coral cover by itself, does not provide information on other important aspects of the community structure and dynamics, or the ecological mechanisms that lead to changes in the coral assemblages (Smith et al., 2005; Grimsditch et al., 2017). Several studies have highlighted the ecological value of complementary metrics to coral cover, such as colony size and their size frequency distribution, to evaluate the condition of the coral communities and reef health (Meesters et al., 2001; Harris et al., 2014; Mumby & Harborne, 2010). In corals, life-history processes, such as reproduction and mortality, are related to size (Meesters et al., 2001) and seem to be affected by subtle environmental changes. Consequently, the size structure of corals is an important driver of the population dynamics (Bak & Meesters, 1998). Size-frequency distributions can help to understand the responses of the population to conditions of local stress or larger scale environmental changes (Hughes & Jackson, 1980; Hughes, 1984). Furthermore, size-frequency data make it possible to analyse ecological processes such as recruitment, fecundity and mortality, in retrospect as well as the potential community responses to disturbances (Meesters et al., 2001; Vermeij & Bak, 2003; Adjeroud, Penin & Carroll, 2007; McClanahan, Ateweberhan & Omukoto, 2008; Gilmour et al., 2013; Turner et al., 2015; Grimsditch et al., 2017).

The conventional methods for recording coral cover are based on collecting the information directly *in situ*, using line transect replicas (Loya, 1972; Porter, 1972; English, Wilkinson & Baker, 1997; Hill & Wilkinson, 2004). Along each transect, systematically distributed points are used to record and count each colony of the coral species and the data are subsequently transformed to obtain cover percentages (e.g., Lang et al., 2010). The standard method to assess size-frequency distributions, is to measure each colony *in situ* with a ruler or a measuring tape (Burgess et al., 2010; Victor et al., 2009; Richardson & Voss, 2005; Smith et al., 2005). Recently, underwater digital photogrammetric techniques and Structure-from-Motion (SfM) algorithms (Longuet-Higgins, 1981; Smith, Carrivick & Quincey, 2015; Carravick, Smith & Quincey, 2016), have been increasingly used for the assessment of coral reef communities across several spatial and temporal scales (Burns et al., 2015a; Burns et al., 2016; Edwards et al., 2017; Rossi et al., 2019). These techniques allow the construction of accurate two (2D) and/or three-dimensional (3D) ortho-mosaics at a very high spatial resolution (few centimetres), which make it possible to characterise the structure of the coral community at scales as small as the size of a coral polyp (Burns et al., 2015a; Burns et al., 2015b; Royer et al., 2018).

In this study we used underwater digital photogrammetry, SfM algorithms and spatial analysis, henceforth referred to as photogrammetry techniques, to assess the live coral cover (m^2^) and to describe the size-frequency distribution of the coral species of six assemblages in shallow reefs of the Cozumel Reefs National Park (CRNP). These reefs are distributed along a north to south gradient of decreasing urban growth and increasing reef development. In addition, we explored the size of the minimum sampling area (m^2^) required to obtain a representative sample of the local species pool (maximum species richness in a minimum area). Cozumel’s reefs are some of the most important reef systems in the Mexican Caribbean and are among the healthiest of the MAR (McField et al., 2018). Our results could provide support to the current monitoring programs in the Mexican Caribbean (and elsewhere in the region) and establish a baseline for long-term surveys based on digital photogrammetry, facilitating the ongoing assessment of local and regional coral reef condition.

## Materials & Methods

### Study area

**Figure 1:**
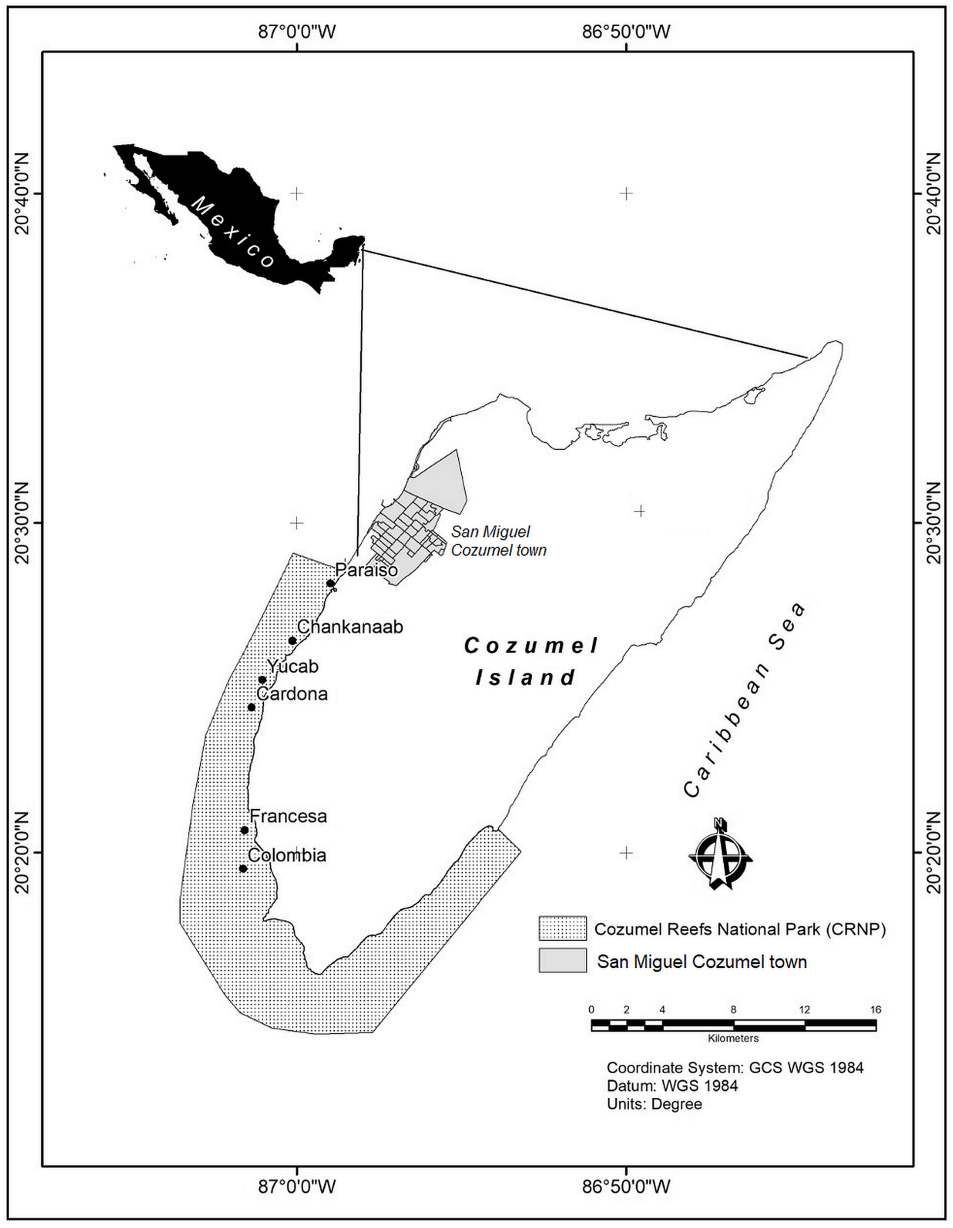
Study area. Cozumel Reefs National Park (CRNP). Reefs sampled from north to south were: Paraiso (PA), Chankanaab (CH), Yucab (YU), Cardona (CA), Francesa (FR) and Colombia (CO).

The Cozumel Reefs National Park (CRNP) is administered by the National Commission of Natural Protected Areas (CONANP), which is the main government institution responsible for the management and conservation of protected areas in Mexico. With an extension of c.a. 12,000 ha, most reefs are located around the south east, south and south west coast of Cozumel Island, in the Mexican Caribbean (Fig. 1). The fringing reefs follow a marked north-south gradient of increasing reef structural complexity and habitat diversity, where larger massive coral structures, mainly of the *Orbicella* complex, can rise several metres above the seafloor (Fenner, 1988; Muckelbauer, 1990; Jordán, 1988; Jordán-Dahlgren & Rodríguez-Martínez, 2003; Rioja-Nieto, Chiappa-Carrara & Sheppard, 2012). The seascape consists of a mixture of sandy beds, fringing reefs, patch reefs, coral colonies on hard substrate, seagrasses and macroalgae (Rioja-Nieto & Sheppard, 2008). The reefs of the CRNP are considered to have the highest coral cover of all the reefs of the MAR (McField et al., 2018). Urban development is concentrated in San Miguel town (Fig. 1), and a few hotels and beach clubs distributed along the southwest of the island. The main economic activities are mostly related to tourism. Local authorities reported that in 2017 c.a. 5 million tourists visited the island, making Cozumel one the most visited islands in the Caribbean (Cruz-Vázquez, Rioja-Nieto & Enríquez, 2019). Cozumel is considered one of the best areas for SCUBA diving, and the marine protected area is heavily visited by people who engage in a variety of recreational activities (Rioja-Nieto & Álvarez Filip, 2018).

### Data collection and construction of ortho-mosaics

Between February and March 2018, six reefs distributed across the western area of the CRNP were visited: Paraiso (PA), Chankanaab (CH), Yucab (YU), Cardona (CA), Francesa (FR) and Colombia (CO) (Fig. 1). The reefs are distributed c.a. 30 kms along the southwest coast of the protected area and were chosen considering their location and their relative proximity to the main urban area (San Miguel Cozumel town). The reefs closest to the town were Paraíso and Chankanaab, while the reefs furthest from the town were Francesa and Colombia. The reefs selected in the central region of the study area were Yucab and Cardona. The sites selected for this study corresponded to the shallow reef front habitat, whose average depth varies from 7.3 m (Cardona and Paraiso) to 14.1 m (Yucab). For each site, transects (*n* = 3) of 30 m in length separated by 20 to 30 m, were placed parallel to the coast following the reef’s development. Along each transect, marks made of polyvinyl (0.6 × 0.6 m), four at the corners and one at the centre of the transect, were placed to delineate a rectangular plot c.a. five m wide (at least 150 m^2^). The plot was divided at the middle mark and photographed by two divers (each focusing on one half of the plot), swimming in a gridded pattern at a constant speed of c.a. 5 m/min. Photographs were taken two metres above the average depth of the reef bottom (Edwards et al., 2017). The depth at the beginning and at the end of each transect was recorded using a standard dive computer. To obtain the photographs, two Canon G12 10-megapixel cameras (Canon WP-DC34) in waterproof housing were used. The settings used were: 4:3 frame size, at a 28 mm default focal length. We set the camera options to self-timer to automatically shoot ten times continuously (1 fps), before taking a new series of images. This ensured a high overlap (>80%) across and along images, which is necessary to construct ortho-mosaics via SfM algorithms (Pix4D, 2019b. https://support.pix4d.com/hc/en-us/sections/200591059-Manual). Pix4Dmapper (4.3.x) software was used to process images and to construct ortho-mosaics from photographs. This software follows three basic steps: (1) initial processing (internal/external camera orientation and sparse cloud creation), (2) point cloud and mesh generation, and (3) Digital Surface Model (DSM) and ortho-mosaic construction. Considering that the images obtained have no geolocation, and it is difficult to obtain accurate ground control points underwater, the marks (or quadrants of 0.60 × 0.60 m) were used as a contrast measurement of length to transform the model to absolute measurements (Pix4D, 2019a. https://support.pix4d.com/hc/en-us/articles/205360375-How-to-scale-a-project). The precision of the ortho-mosaic constructed was between 0.0015 and 0.0067 m of the computed length error. Ortho-mosaics were exported to ArcGIS 10.6 (Environmental Systems Research Institute ESRI, 2017) for analysis. The sections for each plot were joined along the central mark, using the spatial adjustment extension. This resulted in one ortho-mosaic for each plot (total *n* = 3 plots per reef). A great effort was made to maintain the width of the plots. However, this varied due to local current conditions and the lack of a visual reference to delimit the sides of the plots. CONANP, Dirección del Parque Nacional “Arrecifes de Cozumel” provided permits (F00.9/DPNAC/360/18) for this study.

### Data analysis

Shapefiles associated with each of the ortho-mosaics were constructed by digitising the area covered with live coral tissue of all the colonies recorded ≥ 3 cm^2^. Data matrices were generated from the identification of species to obtain the colonies’ abundance, species richness (*S*) and cover (cm^2^) of each site. The species were identified following the criteria of the AGRRA protocol (Lang et al., 2010; Kramer, 2003) and the Human & Deloach (2002) keys. In accordance with Loya (1972), a coral colony was considered as a set of polyps interconnected by live tissue, detached and growing regardless of neighbouring colonies. When coral colonies presented portions clearly separated without live tissue and/or the calcareous skeleton was visibly eroded or overgrown by another benthic group (e.g., macroalgae), every living portion was considered as an independent colony. In the cases where corals showed growth in patches or large clumps (e.g., *Porites porites* and/or *Agaricia tenuifolia*), extreme care was taken to digitise the living tissue, avoiding the dead areas of the colony in order to consider these clumps as one single colony.

To assess whether the areas sampled at each reef were large enough to register a representative local species pool, accumulation curves were estimated (Moreno & Halffter, 2000; Moreno & Halffter, 2001; Ma, Tarmi & Helenius, 2002; Chittaro et al., 2010). For each plot, the number of colonies per cell on a 1 × 1 m mesh was obtained by using scripts written in Matlab and an envelope procedure known as Convex Hull (Jarvis’s or Wrapping Algorithm, see Graham, 1972; Andrew, 1979; Kirkpatrick & Seidel, 1986). Community matrices of species richness and abundance were then constructed. EstimateS V9.10 (Colwell, 2013), was used to generate species accumulation curves and to obtain the corresponding Shannon diversity values (*H*′), based on 100 randomised iterations without replacement (Gotelli & Colwell, 2011; Gotelli & Chao, 2013). The curves were subjected to increases of 25 m^2^ (up to the maximum area sampled in each site), using a non-parametric Bootstrap procedure. A Bootstrap estimator was chosen as it proved to be the most precise of the seven estimators given by default in the Estimate software during previous tests. The Bootstrap estimator was the least biased (low mean error -ME-), with the highest precision (low variance -VAR-) and accuracy (low mean standard error -MSE-). The performance of the estimators was evaluated following the criteria of Hellmann & Fowler (1999) and Walther & Moore (2005). A Chi-Square goodness-of-fit test (*χ*^2^, *p* = 0.05) was used to compare the observed richness against the richness estimated by Bootstrap for each reef. Spearman’s rank coefficients (*r*_*s*_), were calculated to correlate (*p* = 0.05) the sampling effort (area in m^2^) and diversity values.

To model the relationship between the sampling effort and the number of species observed, the data were fitted to the asymptotic Clench model (Jiménez-Valverde & Hortal, 2003; Gómez-Anaya et al., 2014). The model equation used to estimate the number of predicted coral species for each reef was: *S*(*x*) = *ax*∕(1 + *bx*), where *x* is a measure of sampling effort, *S*(*x*) is the predicted number of species at effort *x*, a represents the rate of increase at the beginning of sampling, *b* is a parameter related to the form of the accumulation curve and *a/b* is the asymptote. To fit the model, the mean number of species per sample was used from community matrices (Colwell, 2013). The model fit was obtained using the non-linear estimation module in STATISTICA V10 (StatSoft, 2011), applying the Simplex and Quasi-Newton methods for parameter estimation. The theoretical effort required (nq) for each inventory was then calculated with the equation: *nq* = *q*∕[*b*⋅(1 − *q*)], where *q* is the relative proportion of the list of species to be detected (Jiménez-Valverde & Hortal, 2003). The first approach to the curve asymptote or the total number of species predicted (calculated as *a*∕*b*) and the coefficient of determination (*R*^2^), which is a descriptive measure of the proportion of explained variance, were also obtained for each reef.

The distribution of the abundance of colonies and live coral cover (m^2^) per reef was analysed and statistically compared by means of similarity analyses (One-way ANOSIM, 9999 permutations, *p* = 0.05). For both parameters, similarity matrices based on the Bray-Curtis coefficient were generated and transformed with the square root (Clarke & Warwick, 1994). Subsequently, the size-frequency percentages (%) of each reef were distributed in 12 size-classes with ranges of 200 cm^2^. The largest size class corresponded to colonies >3,800 cm^2^. Non-parametric Mann–Whitney *U* test analyses (*p* = 0.05, 95% of confidence, STATISTICA V10, StatSoft, 2011) were performed to compare the size-frequency distributions between reefs. The data were logarithmically transformed prior to analysis.

SIMPER analyses (Clarke & Warwick, 1994) were performed to identify the coral species that together contributed 90% of the abundance and total live coral cover. A group of twelve species was identified, which in combination formed the dominant species assemblages of each reef. The analyses were performed in PRIMER 6.1.13 and PERMANOVA+ (Clarke & Gorley, 2006). The size-frequencies of the dominant species were subsequently plotted in 12 size classes (cm2) (see Fig. S1 for the size-class ranges for each species). Colony size data were logarithmically transformed, analysed graphically and compared statistically. In accordance with Bak & Meesters (1997) and Bak & Meesters (1998), the logarithmic transformation of coral-size data produces a clear representation of population structure in scleractinian corals. Size distributions of transformed data have a better resolution and reflect an approximate size-frequency distribution more closely than distributions of non-transformed data. Size-distributions were statistically contrasted via one-way non-parametric Kruskal-Wallis analyses (*H* test, *p* = 0.05, 95% of confidence) and subsequently significant differences in the size frequency distribution between reefs were obtained from post-hoc tests based on pairwise comparisons using Mann–Whitney (*p* = 0.05, 95% of confidence). A set of parameters related to the shape of the size-frequency distributions was analysed. Mainly the Skewness (g1) and the Kurtosis (g2), among other distribution parameters (such as, maximum size, 95th percentile and central tendency measures), were explored to describe the colony-size distributions of the dominant species population (Sokal & Rohlf, 1995; Zar, 1999).

**Figure 2:**
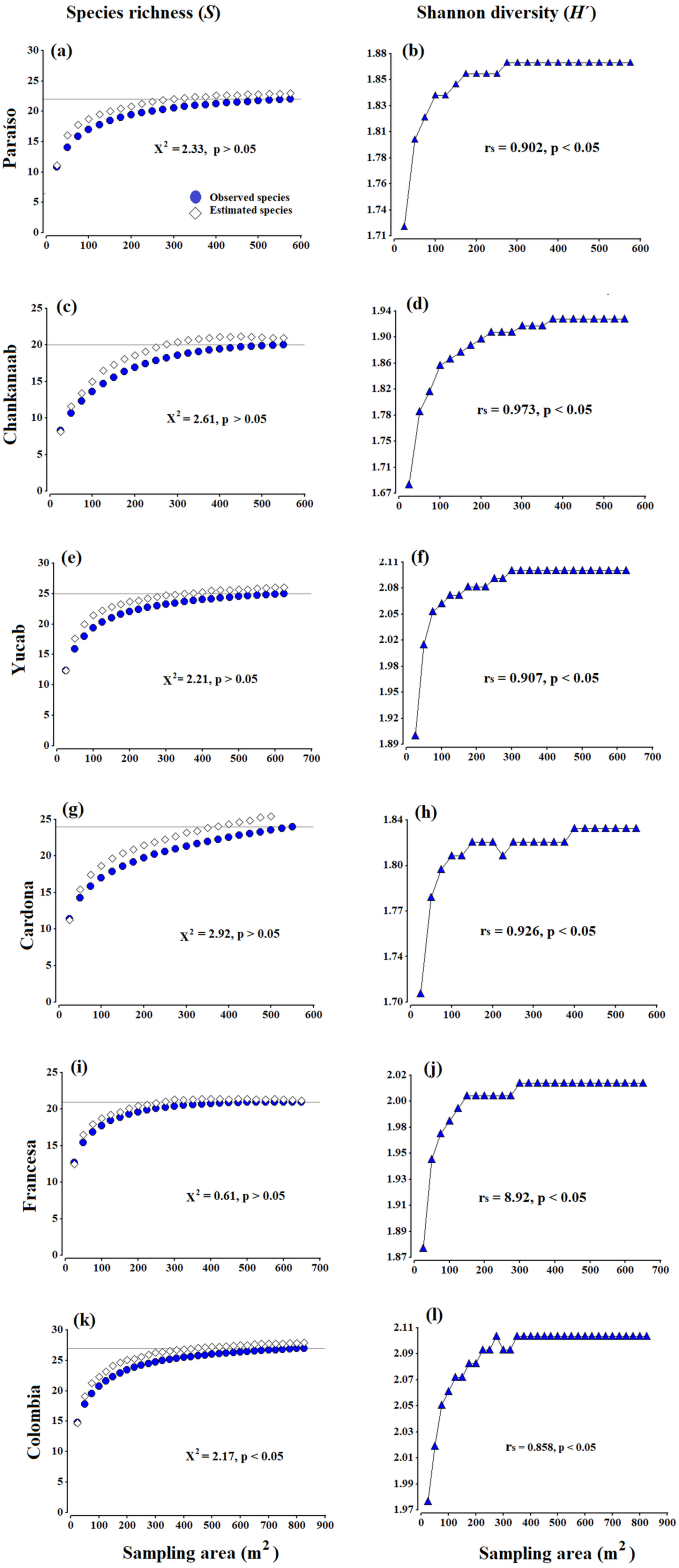
Accumulation species curves. Observed species *vs* estimated species (A, C, E, G, I, K) and Shannon diversity index values (*H*′) (B, D, F, H, J, L), obtained from the Bootstrap estimator. A Chi-square goodness-of-fit test was used to compare the observed richness against estimated richness (*X*^2^, *p* = 0.05, 95% confidence). The Spearman correlation coefficent (*r*_*s*_) (*p* = 0.05, 95% confidence) was used to correlate the sampling effort (area in m^2^) and Shannon diversity index values (*H*′). The grey horizontal line represents the total number of species observed in each site.

## Results

There were no significant differences (*X*^2^ test, *p* > 0.05) between the observed and estimated species obtained from the Bootstrap estimator (Figs. 2A, 2C, 2E, 2G, 2I and 2K). The diversity (*H*′ index values) (Figs. 2B, 2D, 2F, 2H, 2J and 2L), was also positively correlated to the increase in the area sampled (Spearman coefficient, *r*_*s*_ > 85%, *p* < 0.05). Both the curves of richness and diversity values increased rapidly from 25 to 150 m^2^. In all cases, the asymptotic phases began c.a. 300 m^2^, until stabilizing almost entirely around 400 m^2^ (Fig. 2). The only exception was Cardona (Fig. 2G), where the curve began to stabilize after 450 m^2^, which seems to be related to the presence of a few rare species, such as *Scolymia sp.,* which was only observed in this reef and *A. palmata*, which was only recorded in Cardona and Colombia. The maximum value of diversity varied from 1.83 (±0.03) to 2.2 (±0.04), between Chankanaab and Colombia, respectively (Table 1). The values of the determination coefficients (*R*^2^ close to 1) and the high predicted species richness (*R* > 90%) indicate a good model fit, which suggests that sampling an area of 300–400 m^2^ is sufficient to obtain a representative and highly reliable record of coral species richness (Table 1). The number of predicted species (*a/b*) was very close to the total species richness observed at each reef (with a difference of less than one species). Using photogrammetry techniques, an average area of 374 m^2^ (standard deviation SD = 48.1 m^2^) is necessary to record 90% of the coral species in the shallow reefs of Cozumel. This is also supported by the accumulation curves of richness and diversity (Figs. 2B, 2D, 2F, 2H, 2J and 2L). In general, the area recommended to record a high percentage of species (e.g., *nq* = 90%), proved to be less than the total area sampled in the surveyed reefs.

**Table 1:** Values obtained from the species accumulation curves according to Clench’s function.

**Reef**	**S_obs_**	***H*′**	***a***	***b***	***R*^2^**	***R* (%)**	**(*a/b*)**	**(*nq*)**
Paraiso	22	1.87	0.7056	0.0290	0. 994	98.8	22.8	309
Chankanaab	20	1.83	0.3980	0.0181	0. 992	98.4	21.9	495
Yucab	25	2.10	0.8092	0.0249	0.995	99.1	25.9	361
Cardona	24	1.93	0.6113	0.0248	0.974	94.9	24.6	362
Francesa	21	2.10	1.1001	0.0300	0.995	99.1	21.6	299
Colombia	27	2.20	0.9639	0.0351	0.983	96.6	27.3	256

**Notes.**

Site sampled (Reef), species observed (Sobs), diversity values (*H*′), *a* and *b* values, determination coefficient (*R*^2^), explained model variance *R* (%), number of predicted species (*a*∕*b*), and the theoretical sampling (*nq*) to capture 90% of the predicted species richness. The theoretical effort (*nq*), was calculated according to the equation: *nq* = *q*∕[*b*(1 − *q*)], where *q* = the relative proportion of the list of species to be detected (Jiménez-Valverde & Hortal, 2003).

### Coral assemblages

**Figure 3:**
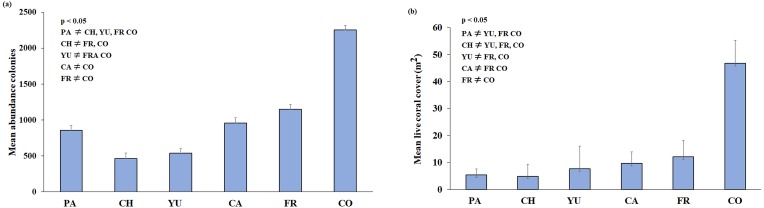
(A) Mean colony abundance and (B) Mean live coral cover. One-way similarity analyses (ANOSIM, 9999 permutations, *P* value = 0.05). Bars represent the Standard Error (±SE). The significant differences between reefs are indicated on the upper left side (*p* < 0.05). Paraiso (PA), Chankanaab (CH), Yucab (YU), Cardona (CA), Francesa (FR) and Colombia (CO).

A total of 3,775 m^2^ (0.38 ha) of reef development was characterised from ortho-mosaics, of which 260 m^2^ (or 6.8% of the total sampled area) was covered by live coral. The precision of the obtained ortho-mosaics was between 0.0015 and 0.0066 m of the computed length error. In total, 18,661 colonies and 32 species of scleractinian corals were recorded (Table 2). The sampling area for each reef ranged from c.a. 550 m^2^ at Chankanaab and Cardona to 825 m^2^ at Colombia reef. The lowest species richness (20) was observed at Chankannab and the highest (27) at Colombia. Colombia also showed the highest abundance of colonies and live coral cover (*p* < 0.05). Chankanaab had the lowest abundance of all the sampled sites (Fig. 3A, *p* < 0.05). The lowest live coral cover was observed at Paraiso, Chankanaab and Cardona (Fig. 3B, *p* < 0.05). In general, a significant increase (*p* < 0.05) in the number of coral colonies and live coral cover is observed from north to south of the study area.

**Table 2:** List of coral species recorded on six reefs of the CRNP. List of coral species recorded on six reefs of the CRNP. Sampling area (m^2^), Species richness (S), Colonies abundance (*n*), Standardised live coral cover (% of total area). The code used is in accordance with the AGGRA protocol (see https://www.agrra.org/training-tools/coral-training/).

	**Paraíso**		**Chankanaab**		**Yucab**		**Cardona**		**Francesa**		**Colombia**	
Sampling area (m^2^)	575		550		625		550		650		825	
Species richness (S)	22		20		25		24		21		27	
Colonies abundance (*n*)	2,572		1,390		1,620		2,867		3,446		6,766	
Standardised live coral cover (% of total area)	2.9		2.5		3.8		5.4		5.7		17.2	
***Spp name/code***	***n***	**m^2^**	***n***	**m^2^**	***n***	**m^2^**	***n***	**m^2^**	***n***	**m^2^**	***n***	**m^2^**
*Agaricia agaricites AAGA*	635	3.14	428	4.53	280	2.92	1,422	9.69	1,218	7.56	1,702	14.6
*Agaricia fragilis AFRA*	1	0.0024			14	0.15			7	0.07	25	0.13
*Agaricia humilis AHUM*	168	0.62	11	0.11	13	0.08	55	0.2	96	0.52	96	0.65
*Agaricia lamarcki ALAM*	6	0.02									13	0.21
*Acropora palmata APAL*							1	0.0021			1	0.01
*Agaricia tenuifoila ATEN*	12	0.23	6	0.34	20	0.37	21	1.24	191	3.35	877	50.88
*Colpophyllia natans CNAT*					1	0.08					3	0.05
*Dendrogyra cylindrus DCYL*											8	0.44
*Diploria labyrinthiformis DLAB*	21	0.25	6	0.17	2	0.07	8	0.18	5	0.04	9	0.2
*Dichocoenia stokesii DSTO*					3	0.01	2	0.01	3	0.03		
*Eusmilia fastigiata EFAS*	63	0.26	97	0.35	58	0.27	99	0.59	255	1.28	98	0.38
*Favia fragum FFRA*	12	0.01	3	0.0048	4	0.0042	42	0.04	30	0.03	26	0.04
*Isophyllia rigida IRIG*					9	0.09	1	0.01	4	0.06	5	0.08
*Mancina areolata MARE*	8	0.03			1	0.0035						
*Montastraea cavernosa MCAV*	80	1.23	77	1.57	104	1.94	104	2.64	58	1.36	127	3.57
*Madracis decactis MDEC*	2	0.0041	1	0.0034	13	0.07	12	0.04	12	0.07	43	0.17
*Meandrina jacksoni MJAC*			2	0.08	2	0.02						
*Meandrina meandrites MMEA*	16	0.31	8	0.17	14	0.4	2	0.04	7	0.05	6	0.07
*Mycetophyllia sp MYCE*	4	0.01							2	0.02	6	0.05
*Orbicella annularis OANN*	47	0.28	31	0.45	28	0.59	195	3.5	215	3.65	716	13.9
*Orbicella faveolata OFAV*	8	0.21	11	0.18	2	0.27	12	1.34	9	0.63	66	4.25
*Orbicella franksi OFRA*	4	0.08	1	0.0031	1	0.02	1	0.03			15	0.76
*Porites astreoides PAST*	713	2.66	245	1.81	317	2.35	295	2.21	102	0.53	1,090	10.47
*Pseudodiploria clivosa PCLI*			3	0.11	9	0.37	5	0.04			7	0.05
*Porites furcata PFUR*					12	0.24	9	0.03	41	0.51	68	1.12
*Porites porites PPOR*	18	0.07	6	0.05	436	9.21	233	2.18	794	13.37	1430	33.31
*Pseudodiploria strigosa PSTR*	26	0.83	4	0.22	15	0.61	16	0.43	17	0.38	25	0.43
*Solenastrea bournoni SBOU*			5	0.0047			2	0.05				
*Scolymia sp SCOL*							1	0.00025				
*Isophyllia sinuosa SINT*	1	0.01									1	0.13
*Siderastrea radians SRAD*	72	0.31	136	0.48	54	0.17	34	0.12	122	0.39	81	0.51
*Siderastrea siderea SSID*	655	5.63	309	4.07	208	2.77	295	4.43	258	2.48	222	3.96

**Figure 4:**
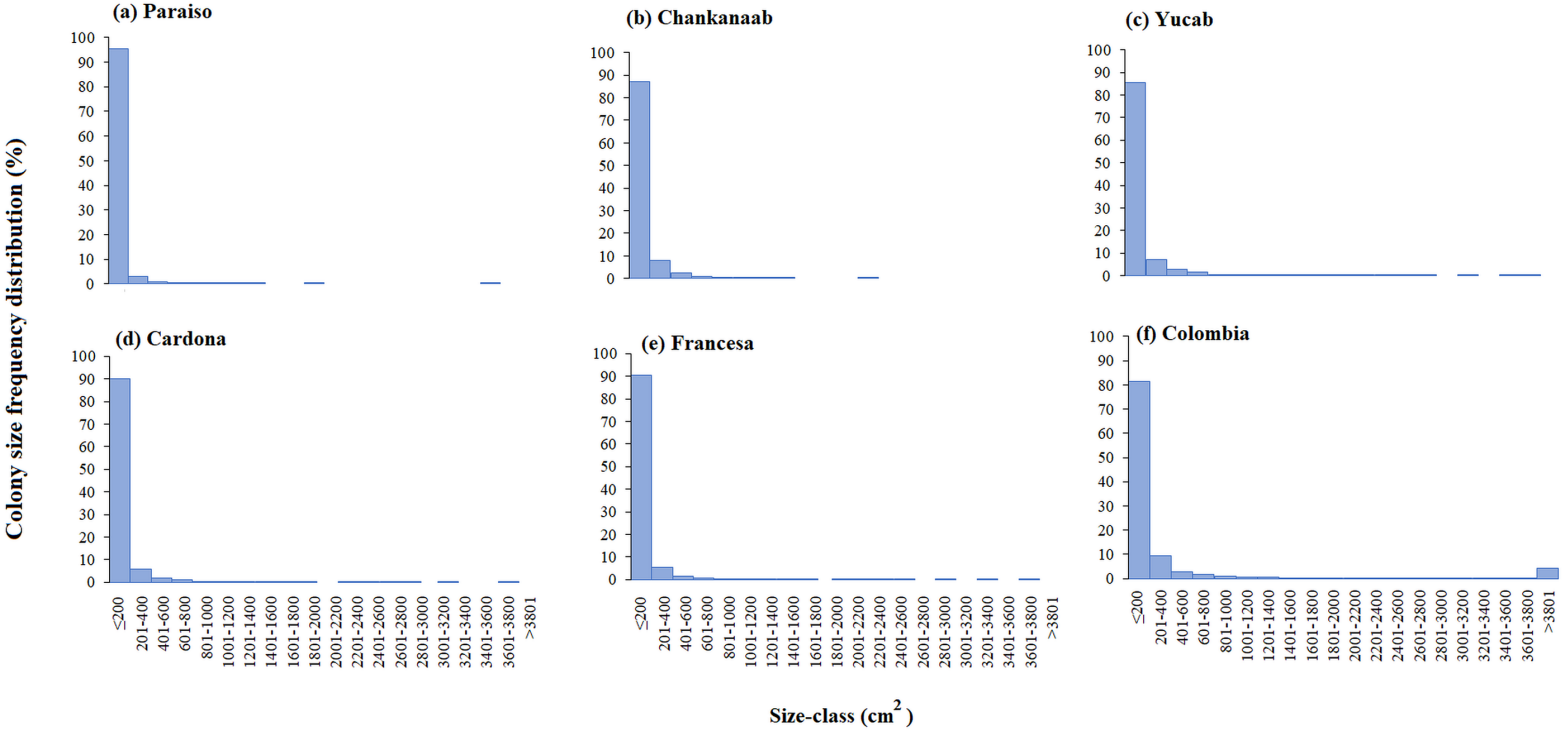
Coral colony size frequency distribution (%) by reef (A-F). Significant differences were found between Chankanaab (B) and Colombia (F) (W = 87.0, *p* < 0.05) and between Yucab (C) and Colombia (F) (W = 8.15, *p* < 0.05).

**Figure 5:**
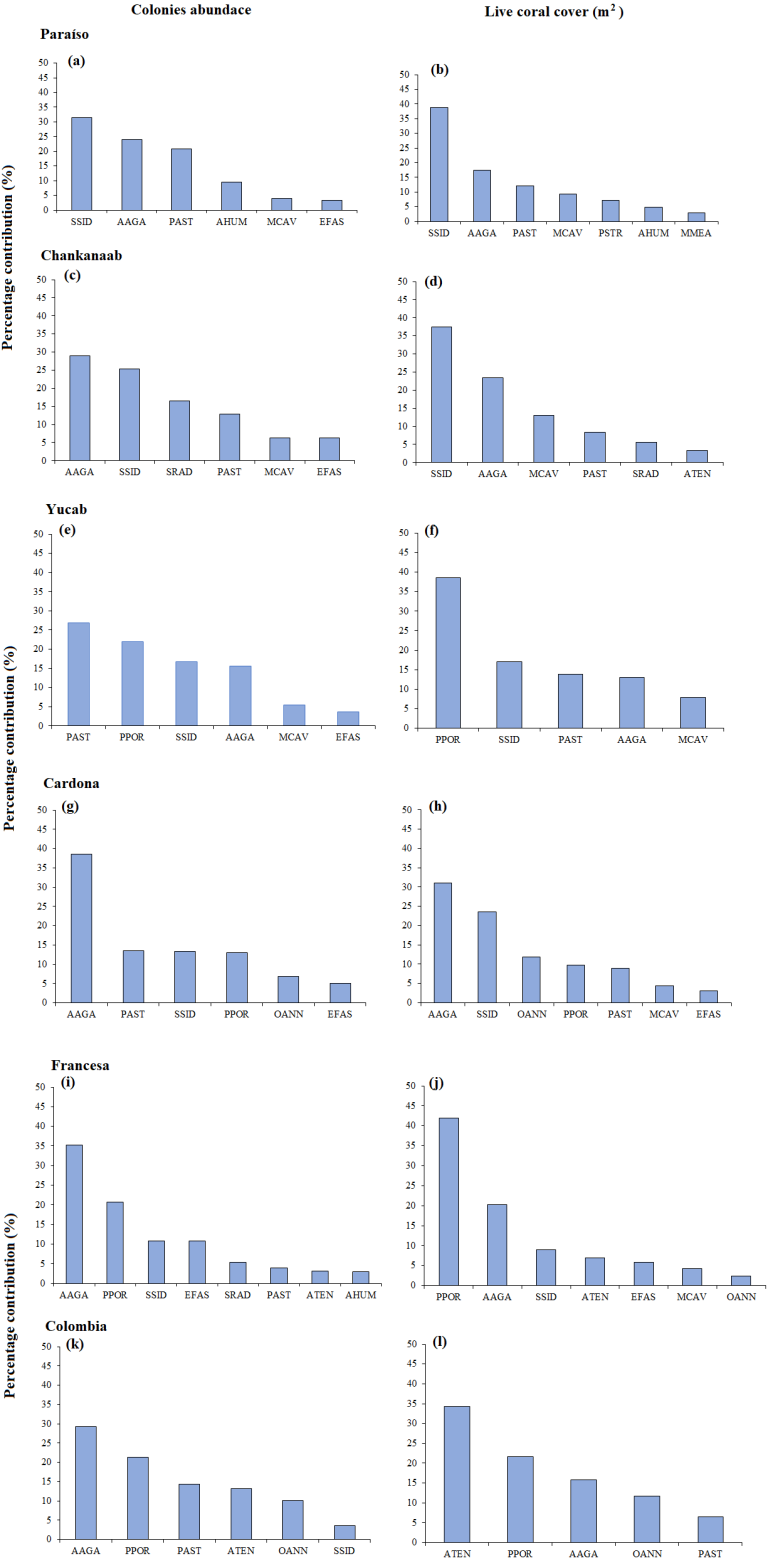
SIMPER analysis. Dominant species assemblages that contributed with 90% of the abundance of colonies (A–K) and live coral cover (m^2^) (B–L) of each reef. *Agaricia agaricites* (AAGA), *A. humilis* (AHUM), *A. tenuifolia* (ATEN), *Eusmilia fastigiata* (EFAS), *Montastrea cavernosa* (MCAV), *Meandrina meandrites* (MMEA), *Orbicella annularis* (OANN), *Porites astreoides* (PAST), *P. porites* (PPOR), *Pseudodiploria strigosa* (PSTR), *Siderastrea radians* (SRAD) and *S. siderea* (SSID).

The size-frequency distribution (Figs. 4A–4F) showed that >80% of the colonies in all reefs were distributed in small size-classes (≤200–400 cm^2^). Significant differences in size distribution were observed between Colombia and Chankannab (*W* = 87.0, *p* < 0.05), and Yucab (*W* = 8.15, *p* < 0.05). A group of twelve species proved to be dominant in terms of abundance (Figs. 5A, 5C, 5E, 5G, 5I and 5K) and live coral cover (Figs. 5B, 5D, 5F, 5H, 5J and 5L). In alphabetical order these were: *Agaricia agarites, A humilis, A. tenuifolia, Eusmilia fastigiata, Meandrina meandrites, Montastrea cavernosa, Orbicella annularis, Porites astreoides, P. porites, Pseudodiploria strigosa, Siderastrea radians* and *S. siderea*. The largest contributions, in terms of abundance, corresponded to sub-massive species, with small and medium sizes, such as *A. agaricites, P. astreoides* and *S. siderea*. In terms of coral cover the greatest contributors were *A. tenuifolia* and *P. porites*, whose foliose and digitiform morphologies tend to form large colony clumps. This dominant coral group represented 34% of the total species richness recorded. *A. agaricites*, was the only species within this group that contributed to both abundance and coral cover at all reefs. *S. siderastrea* and *P. astreoides* contributed in terms of abundance at all reefs, but not in terms of coral cover for Colombia and Francesa. *M. meandrites* and *P. strigosa* were only present in the dominant group of live coral cover at Paraíso. *O. annularis*, an important reef-building coral species for Caribbean reefs, was only present in the dominant species group in terms of abundance and/or coral cover at Cardona, Francesa and Colombia. Species considered as important reef-building corals (e.g., *O. faveolata* or *A. palmata*), in general showed very low contributions (<5%).

The number of colonies recorded by species and size-frequency distribution (non-transformed size data) in twelve independent size-classes for each species are shown in Fig. S1. A wide intraspecific variation between reefs was observed. In general, the colonies were concentrated in the smallest size-classes and declined markedly towards the largest size-classes. Abundant living colonies ≥3 cm^2^ (viable fragments or small recruits) were recorded for several species such as, *A. tenuifolia*, *E. fastigiata, P. porites*, *S radians* and *S. siderea*. The largest colonies recorded corresponded to *P. strigosa* (2,135 cm^2^), *M. cavernosa* (3,176 cm^2^), *O. annularis* (4,670 cm^2^), *P. astreoides* (8,478 m^2^), *P. porites* (14,378 cm^2^) and *A. tenuifolia* (17,106 cm^2^) (see Table S1).

**Figure 6:**
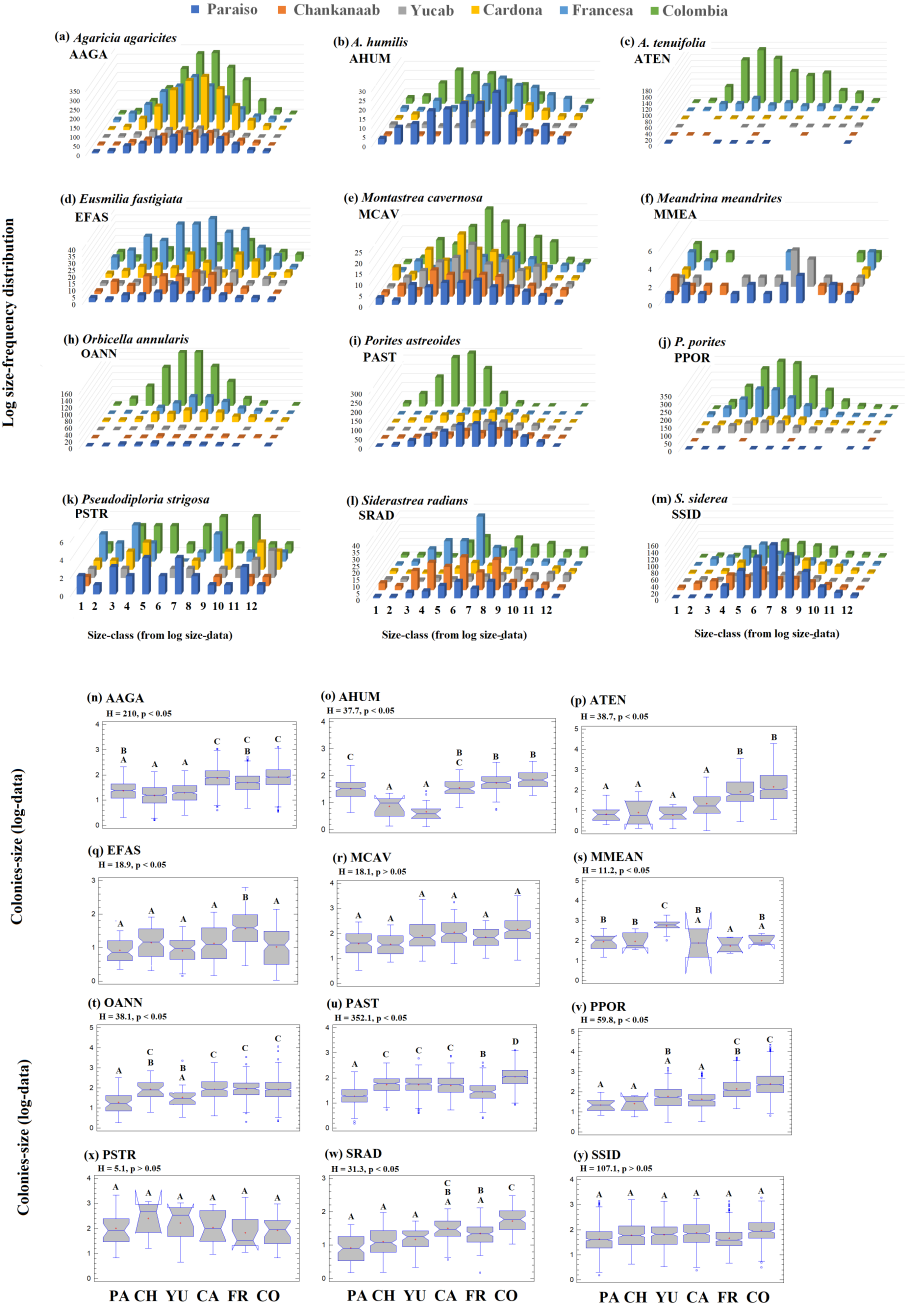
Frequency-distribution. Log size-frequency distribution of the twelve dominant species (A–M) and intraspecific contrast between of the colonies-size distribution by reefs (N-Y). Different letters indicate significant differences (Kruskal-Wallis *H* test, *p* = 0.05, 95% confidence). Sites: Paraíso (PA), Chankanaab (CH), Yucab (YU), Cardona (CA), Francesa (FR) and Colombia (CO). The size classes (1–12), correspond to the class ranges of Fig. S1. *Agaricia agaricites* (AAGA), *A. humilis* (AHUM), *A. tenuifolia* (ATEN), *Eusmilia fastigiata* (EFAS), *Montastrea cavernosa* (MCAV), *Meandrina meandrites* (MMEA), *Orbicella annularis* (OANN), *Porites astreoides* (PAST), *P. porites* (PPOR), *Pseudodiploria strigosa* (PSTR), *Siderastrea radians* (SRAD) and *S. siderea* (SSID).

The 95th-percentile size was used as a distribution parameter to determine the maximum colony size of the coral population (see Soong, 1993; Meesters et al., 2001). In this study, the colony size at the 95th-percentile varied considerably for the same species between sites, even for adjacent reefs (Table S1). For example, in the case of *A. agaricites*, the maximum colony size at the 95th percentile was twice as large at Chankanaab as in Paraiso (326.2 cm^2^ and 141.5 cm^2^, respectively). For other species, such as *A. tenuifolia,* the maximum colony size at the 95th percentile varied up to an order of magnitude, with 393.9 cm^2^ at Yucab and almost 3,000 cm^2^ at Colombia (see Table S1). The size data were logarithmically transformed (Soong, 1993; Bak & Meesters, 1997; Bak & Meesters, 1998), analysed graphically and compared statistically. The distribution of the size-frequency of the dominant species is shown in Figs. 6A–6M, and the probability of the data being normally distributed (Kolmogorov–Smirnov normal test using -KS-, *p* = 0.05), is shown in Table S1. The frequency distribution parameters indicated that the size-distribution of the species was not symmetrical around the mean. The species of the dominant group, *A. humilis, E. fastigiata, P. astreoides, P. strigosa* and S. radians showed mainly a negative skewness for most reefs. Those that presented mainly positive skewness were *A. tenuifolia, O. annularis, P. porites* and *S. siderea*. Others, such as *E. fastigiata, P strigosa, S. radians*, and *M. meandrites* presented distributions with skewness that were highly variable between reefs (Figs. 6A–6M and Table S1). A unidirectional tendency to shift from negative to positive skewness from north to south across the study area was observed for *P. astreoides.* Predominantly negative or platykurtic kurtosis was observed in the size-distributions of *A. agaricites, E. fastigiata, M. cavernosa* and *P. strigosa* across all reefs (Table S1). The colonies’ size range (Figs. 6N–6Y) increased in a north-south direction (*p* < 0.05) for *A. agaricites, A. humunis, A. tenuifolia, O. annularis, P. astreoides* and *P. porites* (Figs. 6N, 6O, 6P, 6T, 6U and 6V, respectively). Significant differences (H test, *p* < 0.05) were also observed in *M. meandrites, E. fastigiata*, and *S. radians.* However, these species showed no size distribution pattern on a latitudinal gradient. *M. cavernosa, P. strigosa and S. siderea* did not present significant differences between reefs. These species were more homogenously distributed among reefs in terms of the median size range (Figs. 6R, 6X and 6Y, respectively).

## Discussion

The colony coral cover (m^2^) and the structure and pattern of the corals’ size-frequency distribution revealed important characteristics of the structure and composition of the coral assemblages in the shallow reefs of Cozumel. The live coral cover (m^2^) quantified from using underwater photogrammetry techniques, resulted in lower percentages when compared to those estimated by the most used conventional methods of observation *in situ* in the Caribbean region. Twelve coral species were significantly dominant in terms of abundance and live cover (in alphabetical order): *Agaricia agarites, A humilis, A. tenuifolia, E. fastigiata, M. meandrites, M.cavernosa, O. annularis, P. astreoides, P. porites, P. strigosa, S. radians* and *S. siderea.* Both the abundance and live coral cover increased along a north-south gradient for some species. Species with the highest number of colonies do not always contribute the largest live coral cover. For example, *A. tenuifolia* was not among the most abundant species on the reefs located to the north of the study area. However, this species was dominant on the reefs located in the south of the protected area, with the highest abundance in Francesa, and highest coral cover in Colombia. In Colombia reef, *A. tenuifolia* and *P. porites* were also determinant in the significant differences observed in live coral, with low abundance but high cover. Unlike other coral reefs, at Colombia, large colonies of these species were recorded forming extensive clumps. For most colonies, the size-frequency of the species ranged from ≥ 3 to around 400 cm^2^, corresponding to the first size-class respectively for each dominant species. A minimum sampling area of c.a. 380 m^2^ is suggested in order to facilitate the use of digital photogrammetry techniques to characterise coral reefs in the Caribbean.

Underwater digital photogrammetry techniques, have recently been applied to map and assess several attributes of coral assemblages, such as cover, condition, growth and structure (Lirman et al., 2007; Westoby et al., 2012; Gintert et al., 2012; Burns et al., 2015b; Edwards et al., 2017; González-Rivero et al., 2017; Gintert et al., 2018). Here, the high resolution of the obtained ortho-mosaics allowed a high number of colonies to be quantified, and the real area covered by live coral in colonies from 3 cm^2^ to be estimated. The coral reefs of the CRNP have been well characterised and monitored since the early 1990’s (e.g., Jordán, 1988; Fenner, 1988; Fenner, 1991; Fenner, 1999; Alvarez-Filip et al., 2009; Alvarez-Filip et al., 2011; Reyes-Bonilla, Millet-Encalada & Álvarez Filip, 2014; McField et al., 2018). The species richness recorded, corresponded to >80% (32) of the species previously reported. When comparing the percentage of live coral cover estimated in this study to recent assessments in the CRNP (see Reyes-Bonilla, Millet-Encalada & Álvarez Filip, 2014; Barranco et al., 2016; Loreto-Viruel et al., 2017; McField et al., 2018), the results were considerably lower. Coral cover ranged between 2.5 and 17.2%, with a mean live cover of 6.2% (±SE = 1.8), where previous studies establish a coral cover between 11 and 25% (average 20%). Considering that no major disturbances (e.g., hurricanes, disease) were observed during sample times and that the characterised reefs are the same used in previous studies, the difference in coral cover seems to be related to the sampling method used. Registering information *in situ* is possibly the most widely used technique to monitor reefs in the Mexican Caribbean (e.g., AGRRA protocol, Healthy Reefs Initiative -HRI, Lang et al., 2010; McField et al., 2018). In comparison, the high-resolution of the ortho-mosaics, allows a more precise quantification of the area covered by living coral tissue (cm^2^). Although substantial post-processing time was required for the analysis of each colony, the live tissue can be estimated from colonies of a few centimetres in size (e.g., fragmented colonies or small recruits) to large adult colonies, thus encompassing a wide range of spatial scales (e.g., a few centimetres to tens or hundreds of metres). The difference observed between the coral cover reported in previous studies from *in situ* estimates (mentioned above), against those obtained here, may suggest an overestimation by the traditional methods. However, it is not easy to determine whether one method or the other results in an underestimation or overestimation of coral cover. This needs to be carefully evaluated through the contrast of cover data acquired from the most commonly used evaluation methods (e.g., transect line), against those obtained by digital photogrammetry, which until now has not been explored.

Locally the species richness and diversity values obtained were representative of each reef studied. This was supported by the observed and estimated species richness obtained via the Bootstrap procedure for each reef. The Bootstrap curves did not differ significantly (*X*^2^, *p* > 0.05) and showed strong and positive correlations (Spearman *r*_*s*_) between the diversity (*H*′ > 1.80) and increase in sampling area. The data fit to the Clench model and the subsequent estimated theoretical effort required (nq), suggested that an average area of c.a 380 m^2^ is appropriate to record at least 90% of the local coral species pool in the shallow reefs of Cozumel, using underwater digital photogrammetry. The area sampled at each reef was larger than the average suggested. Therefore, the observations are reliable to assess the species richness, live coral cover and the colony size structure of the coral assemblages of the shallow reefs in the CRNP. Considering that in recent years, the use of photogrammetry techniques (from digital photography and video) in underwater monitoring has increased, the estimation of an average sampling area to record a representative sample of the local coral richness, is relevant. This should help to improve the efficiency of future ecological sampling (e.g., significantly reduce work in the field, permit more sites to be surveyed, save time collecting data, reduce bottom-time underwater, save money, among others) (Lirman et al., 2007; Burns et al., 2015a; Burns et al., 2015b). The average minimum sampled area (m^2^) estimated in this study, could be applied to reefs of similar characteristics in the Caribbean, for example in terms of the depth range and reef-development.

A complementary monitoring program based on digital photogrammetry could allow changes in live coral cover area, colony growth, mortality, and recruitment of coral species and other important benthic groups to be described at a resolution of centimetres over extensive areas. In this study, the assessments were performed on six shallow reefs at different depths (between 6 and 14 m). Fringing shallow reefs border most of the continental and insular shores of the Mexican Caribbean and are common in other areas of the Caribbean Sea (Jordán-Dahlgren & Rodríguez-Martínez, 2003). Therefore, our results could be considered as representative of fringing shallow reefs in the region. In the CRNP, high species richness, a large number of colonies, and a wide range of coral colony sizes were obtained using photogrammetry. These are key components for coral populations demographic studies and the determination of general reef health (Meesters et al., 2001; Lang et al., 2010; Mumby & Harborne, 2010; McField et al., 2018). The results highlighted that the minimum and maximum size recorded for a single species varied substantially even when the reefs were located next to one another. In general, the greatest size-frequencies (>80%), were distributed among small size classes ≥3 to ≤400 cm^2^. Most of the frequency distribution of the species between reefs had a positive skewness, which is considered as proxy of greater abundance of young colonies and a constant input of coral recruits (Bythell, Bythell & Gladfelter, 1993; Soong, 1993; Lewis, 1997; Meesters et al., 2001; Kenyon et al., 2006). The predominance of negative kurtosis or platykurtic (non-peaked distribution) and wide standard deviations observed in the dominant species of this study, are also indicative of a high variation in colony size and suggest stable coral populations in demographic terms. In contrast, the shape of the size-distributions of a similar group of species in degraded reefs, were negatively skewed, with extremely positive kurtosis, suggesting limited recruitment and a low frequency of large adult colonies (see Meesters et al., 2001).

Other parameters used to describe the size-frequency distribution, such as the mean colony size, provide information about coral life-history strategies, such as growth and reproduction (Soong & Lang, 1992). The wide intraspecific variation between reefs observed, was in accordance with other studies where variations in the mean size between species and reef sites were up to an order of magnitude. The maximum colony size (based on the 95th percentile), has been correlated with reproductive traits such as the size at reproductive maturity of many conspicuous coral species of the Caribbean region (Soong, 1993). Previous studies have demonstrated that different species reach maturity at different sizes (Szmant-Froelich, Reutter & Riggs, 1985; Szmant, 1986; Rinkevich & Loya, 1986; Babcock, 1984; Babcock, 1991; Pareja-Ortega & Quan-Young, 2016). In small species such as *S. radians*, puberty starts c.a. 10 cm^2^, and in medium size species, such as *M. cavernosa*, it starts at 20 cm^2^ and for *P. astreoides* and *S. siderea* between 70–100 cm^2^ (Soong, 1993; Meesters et al., 2001).

Here, the mean colony size of the species mentioned above comparatively exceeds the estimated size of reproductive maturity, which suggests that populations are in a healthy state of development. The reproductive strategies of corals and the habitat characteristics with respect to colony size, are decisive in the structuring of coral populations (Szmant, 1986; Hall & Hughes, 1996). The reproductive stage of small and medium size species starts early, they brood larvae and have relatively high rates of recruitment, whereas the large species are reproductively mature much later. When large sizes are reached, then these species release gametes during spawning events (Szmant, 1986; Richmond & Hunter, 1990; Hall & Hughes, 1996). Likewise, colony mortality rates have been inversely related to colony size (e.g., Hughes & Jackson, 1980; Hughes & Jackson, 1985; Hughes & Connell, 1987). The larger colonies are more susceptible to partial mortality than smaller colonies (Soong & Lang, 1992; Soong, 1993; Meesters, Wesseling & Bak, 1997). High partial colony mortality of large colonies has a negative effect on the larger size classes by decreasing their proportions and simultaneously increasing the proportion of colonies in the medium-sized classes. In addition, the fission or fragmentation of large colonies due to high physical stress can substantially increase the number of colonies in small-sized classes (Hughes & Jackson, 1980; Jaramillo-González & Acosta, 2009). The implications of these processes on the demographic dynamics of coral populations have been difficult to determine and have not yet been quantified in detail, primarily because of their complicated modular construction and growth. The partial mortality, fission and colony fusion can confuse any simple relationship between the size and age of the corals and mask the origin of individuals in size distribution analysis (Hughes & Jackson, 1980).

The ecological features found in this study, that relate to the size-frequency distribution of the dominant species of the shallow reefs in the CRNP, agree with the distributions described above for small and medium species. Most of these species (e.g., *A. agaricites, P. astreoides, M. cavernosa, O. annularis* and *S. siderea*) lead the current hierarchical shifts in species dominance in the coral assemblages of the Caribbean reefs (Reyes-Bonilla, Millet-Encalada & Álvarez Filip, 2014; Barranco et al., 2016; González-Barrios & Alvarez-Filip, 2018; Estrada-Saldívar et al., 2019), and are considered to have a limited capacity to substantially contribute to the reef framework (Alvarez-Filip et al., 2011; Graham & Nash, 2013; González-Barrios & Alvarez-Filip, 2018). Nevertheless, the population characteristics of the dominant species in terms of their contribution to abundance, live coral cover and size-frequency distributions, imply healthy, stable and potentially resilient coral populations (Loya, 1976; Connell, 1978; Soong, 1993; Bak & Meesters, 1998; Meesters et al., 2001; Richardson & Voss, 2005; Grimsditch et al., 2017).

A wide intraspecific variation among reefs was identified in terms of the size-frequency distribution. However, mean abundance and live coral cover and colony size distribution, increased from north to south for most of the dominant species, which might be related to anthropogenic disturbance. Numerous studies have documented the presence of fewer colonies of small size classes in reefs closer to heavily urbanized coastal areas, with high levels of pollution, sediment load and fishing pressure (Meesters et al., 2001; Vermeij & Bak, 2003; Adjeroud, Penin & Carroll, 2007; McClanahan, Ateweberhan & Omukoto, 2008; Crabee, 2009). Colonies of smaller species which grow faster and have shorter generation times, apparently find relatively acceptable conditions for their development and persistence in unstable environments (Meesters, Wesseling & Bak, 1997). On the other hand, some species, such as *S. siderea* and *P. strigosa*, and particularly *M. cavernosa*, showed similar patterns of size-frequency distribution in all reefs. These species seem to be less sensitive and /or better adapted than other species to local stress factors, which could be related to the pressure level and proximity to urban development sources that potentially influence the intraspecific variation observed (Rogers, 1990; Guzman & Holst, 1993; Guzmán, Burns & Jackson, 1994; Meesters et al., 2001). These species could play an important role as wide scale indicators in tracking changes in current coral communities.

## Conclusions

The size frequency distribution of coral species has rarely been studied in the field due to the underwater technical requirements and huge effort required to collect colony size data in large areas. The ortho-mosaics obtained from photogrammetry provide repeatable, high resolution measurements that can be used to track coral assemblages in the short term at very fine (cm) scales. We conclude that new complementary tools and simple metrics need to be included in current evaluation programs in order to detect short-term changes in coral assemblages. The suggested average sampling area of c.a 380 m^2^ can help in terms of logistic resources in future evaluations of coral communities using photogrammetry techniques. Likewise, it is suggested that this minimum sampling area is applicable to shallow reefs in the Caribbean region with similar environmental characteristics (depth range and reef-development). However, the minimum area suggested for shallows reefs must be flexible when a deeper, speciose or complex reef is the target. The underwater photogrammetric techniques applied here, revealed important features of the coral assemblages of the shallow reefs in the CRNP. Small, non-reef-building coral species dominate and a wide range of intraspecific variation between reefs with respect to the abundance, live coral and in particular the size-frequency distributions was observed. Local environmental factors seem to act differentially from north to south of the study area, with respect to their proximity to the main urban development. The shape and parameters of the frequency distributions of most dominant species exhibited positive skewness and negative kurtosis, which in addition to the coral cover and colony abundance suggest that the coral populations are stable. However, the frequency of the colonies was mainly limited to small size classes with few large colonies, and reef building species were very scarce. Potential changes in the organization of current coral species can be expected in the short term, related to a relatively new outbreak of a coral disease locally known as “White syndrome”. When this study was carried out, coral diseases were not observed. In the CRNP, White syndrome disease was reported for the first time late in the summer of the same year. This disease has rapidly caused the death of a wide variety of coral species. It has been estimated that main reef-building species have been affected (more than 20 coral species), with live coral cover decreasing dramatically (up to c.a. 40% less live coral in some coastal reefs in the Mexican Caribbean) (Technical report, Cozumel reefs situation, Parque Nacional Arrecifes de Cozumel, CONANP, 2019). In order to obtain robust estimates of community change in terms of live coral cover (m^2^) and size-structure over time, digital photogrammetry techniques should be used as complementary tools to traditional methods.

##  Supplemental Information

10.7717/peerj.8957/supp-1 Figure S1 Intraspecific size-frequency distribution (non-transformed size data) between reefs Number of colonies sampled of each specie (*n*). The size-frequencies were distributed in twelve independent size-classes (cm^2^) for each species. The size class ranges were obtained from averaging the minimum and maximum sizes (cm^2^) recorded for each species, class and reef, respectively. The first and last classes were: for *Agaricia agaricites (AAGA)* (3-72) and (766-1154), *A. humilis* (AHUM) (7-33) and (292-882), *A. tenuifolia* (ATEN) (14-38) and (4051-1710), *Eusmilia fastigiata* (EFAS) (3-32) and (347-646), *Montastrea cavernosa* (MCAV) (6-198) and (2121-3176), *Meandrina meandrites* (MMEA) (36-71) and (429-798), *Orbicella annularis* (OANN) (4-309) and (3366-4370), *Porites astreoides* (PAST) (4-156) and (1677-8478), *Porite porites* (PPOR) (5-503) and (5485-14378), *Pseudodiploria strigosa* (PSTR) (9-119) and (1220-2135), *Siderastrea radians* (SRAD) (3-22) and (221-239), and *S. siderea* (SSID) (3-133) and (1435-1564).

10.7717/peerj.8957/supp-2 Table S1 Distribution parameters of the twelve dominant species. Non-transformed data (cm^2^) Standard deviation (SD), minimum (min) and maximum (Max) colony-size and 95th percentile. Log Data: skewness (g1), kurtosis (g2) and Pnorm (Kolmogorov-Smirnov test). Reefs: Paraiso (PA), Chankanaab (CH), Yucab (YU), Cardona (CA), Francesa (FR) and Colombia (CO). Species code: *Agaricia agaricites* (AAGA), *A. fragilis* (AFRA), *A. humilis* (AHUM), *A. lamarcki* (ALAM), *Acropora palmata* (APAL), *A. tenuifoila* (ATEN), *Colpophyllia natans* (CNAT), *Dendrogyra cylindrus* (DCYL), *Diploria labyrinthiformis* (DLAB), *Dichocoenia stokesii* (DSTO), *Eusmilia fastigiata* (EFAS), *Favia fragum* (FFRA), *Isophyllia rigida* (IRIG), *Mancina areolata* (MARE), *Montastraea cavernosa* (MCAV), *Madracis decactis* (MDEC), *Meandrina jacksoni* (MJAC), *Meandrina meandrites* (MMEA), *Mycetophyllia sp* (MYCE), Orbicella annularis (OANN), *O. faveolata* (OFAV), *O. franksi* (OFRA), *Porites astreoides* (PAST), *Pseudodiploria clivosa* (PCLI), *Porites furcata* (PFUR), *P. porites* (PPOR), *Pseudodiploria strigosa* (PSTR), *Solenastrea bournoni* (SBOU), *Scolymia sp* (SCOL), *Isophyllia sinuosa* (SINT), *Siderastrea radians* (SRAD), *S. siderea* (SSID).

10.7717/peerj.8957/supp-3 Data S1 Raw data The coral cover data in m2 (reef name, plot number, colony number, Matlab code, and LCC/m2 is coral cover). The subsequent spreadsheets contain the community matrices for each reef. The first column refers to the area sampled (m2) and the corresponding rows are the abundance values for each species in the sampled area.

## Additional Information and Declarations

### Field Study Permissions

The following information was supplied relating to field study approvals (i.e., approving body and any reference numbers): Comisión Nacional de Áreas Naturales Protegidas, Dirección del Parque Nacional Arrecifes de Cozumel provided permits (F00.9/DPNAC/360/18) for this study.

### Data Availability

The following information was supplied regarding data availability: The raw data are available in the Supplemental Files.
