# Supplementary figures and images for: Size-frequency distribution of coral assemblages in insular shallow reefs of the Mexican Caribbean using underwater photogrammetry
Source: PeerJ. 2020 Apr 17;8:e8957. doi: 10.7717/peerj.8957 (PMC7169971; doi:10.7717/peerj.8957)

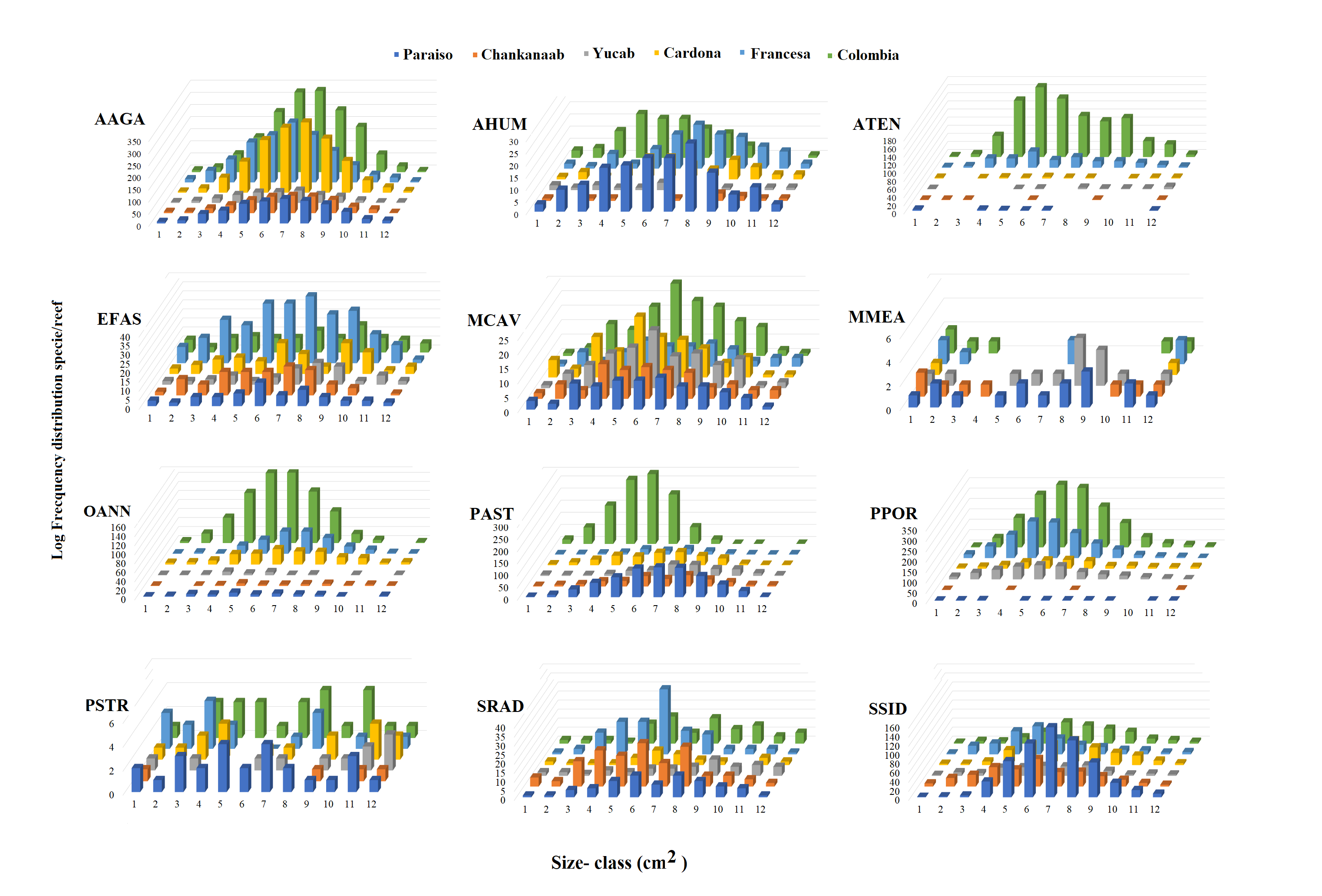

Supplement: Figure S1 — Number of colonies sampled of each specie (n). The size-frequencies were distributed in twelve independent size-classes (cm2) for each species. The size class ranges were obtained from averaging the minimum and maximum sizes (cm2) recorded for each species, class and reef, respectively. The first and last classes were: for Agaricia agaricites (AAGA) (3-72) and (766-1154), A. humilis (AHUM) (7-33) and (292-882), A. tenuifolia (ATEN) (14-38) and (4051-1710), Eusmilia fastigiata (EFAS) (3-32) and (347-646), Montastrea cavernosa (MCAV) (6-198) and (2121-3176), Meandrina meandrites (MMEA) (36-71) and (429-798), Orbicella annularis (OANN) (4-309) and (3366-4370), Porites astreoides (PAST) (4-156) and (1677-8478), Porite porites (PPOR) (5-503) and (5485-14378), Pseudodiploria strigosa (PSTR) (9-119) and (1220-2135), Siderastrea radians (SRAD) (3-22) and (221-239), and S. siderea (SSID) (3-133) and (1435-1564). [file peerj-08-8957-s001.png]
