# Supplementary material for: Size-frequency distribution of coral assemblages in insular shallow reefs of the Mexican Caribbean using underwater photogrammetry
Source: PeerJ. 2020 Apr 17;8:e8957. doi: 10.7717/peerj.8957 (PMC7169971; doi:10.7717/peerj.8957)
Supplement: Table S1 — Standard deviation (SD), minimum (min) and maximum (Max) colony-size and 95th percentile. Log Data: skewness (g1), kurtosis (g2) and Pnorm (Kolmogorov-Smirnov test). Reefs: Paraiso (PA), Chankanaab (CH), Yucab (YU), Cardona (CA), Francesa (FR) and Colombia (CO). Species code: Agaricia agaricites (AAGA), A. fragilis (AFRA), A. humilis (AHUM), A. lamarcki (ALAM), Acropora palmata (APAL), A. tenuifoila (ATEN), Colpophyllia natans (CNAT), Dendrogyra cylindrus (DCYL), Diploria labyrinthiformis (DLAB), Dichocoenia stokesii (DSTO), Eusmilia fastigiata (EFAS), Favia fragum (FFRA), Isophyllia rigida (IRIG), Mancina areolata (MARE), Montastraea cavernosa (MCAV), Madracis decactis (MDEC), Meandrina jacksoni (MJAC), Meandrina meandrites (MMEA), Mycetophyllia sp (MYCE), Orbicella annularis (OANN), O. faveolata (OFAV), O. franksi (OFRA), Porites astreoides (PAST), Pseudodiploria clivosa (PCLI), Porites furcata (PFUR), P. porites (PPOR), Pseudodiploria strigosa (PSTR), Solenastrea bournoni (SBOU), Scolymia sp (SCOL), Isophyllia sinuosa (SINT), Siderastrea radians (SRAD), S. siderea (SSID). [file peerj-08-8957-s002.docx]

|  |  | **Non-transformed data** | | | | |  | **Log Data** | | |
| --- | --- | --- | --- | --- | --- | --- | --- | --- | --- | --- |
| **Specie** | **Reef** | **Mean** | **DE** | **Mín** | **Max** | **P (95)** |  | **g_1_** | **g_2_** | **P normal** |
| ***AAGA*** | PA | 49.5 | 46.7 | 3.0 | 309.7 | 141.5 |  | -0.900 | -0.450 | 0.0715 |
|  | CH | 105.9 | 117.1 | 3.2 | 971.9 | 326.2 |  | -0.230 | -0.140 | 0.0613 |
|  | YU | 104.3 | 116.6 | 5.8 | 1019.7 | 309.3 |  | 0.003 | -0.330 | 0.1049 |
|  | CA | 68.1 | 76.8 | 2.3 | 824.3 | 201.9 |  | 0.010 | -0.150 | 0.0513 |
|  | FR | 62.1 | 71.9 | 3.9 | 726.4 | 191.9 |  | 0.180 | -0.150 | 0.1682 |
|  | CO | 85.8 | 98.3 | 2.3 | 1153.7 | 270.7 |  | 0.160 | -0.190 | 0.0657 |
| ***AHUM*** | PA | 37.0 | 33.1 | 3.5 | 193.1 | 114.2 |  | -0.060 | -0.530 | 0.498 |
|  | CH | 103.9 | 67.8 | 14.4 | 236.9 | 236.9 |  | -0.860 | -0.660 | 0.0187 |
|  | YU | 60.2 | 66.3 | 11.0 | 223.9 | 223.9 |  | 0.620 | -0.720 | 0.0795 |
|  | CA | 36.2 | 26.5 | 5.2 | 132.8 | 86.5 |  | -0.050 | -0.450 | 0.1196 |
|  | FR | 54.2 | 42.1 | 4.2 | 236.1 | 139.7 |  | -0.420 | 0.120 | 0.1948 |
|  | CO | 67.7 | 97.3 | 5.8 | 881.6 | 184.2 |  | 0.300 | 0.370 | 0.0975 |
| ***ATEN*** | PA | 195.6 | 277.6 | 36.5 | 1049.4 | 1049.4 |  | 0.850 | 0.430 | 0.0013 |
|  | CH | 572.1 | 848.6 | 35.1 | 2213.0 | 2213.0 |  | 0.490 | -1.290 | 0.0001 |
|  | YU | 182.7 | 131.1 | 7.0 | 413.6 | 393.9 |  | -1.190 | 1.120 | 0.0276 |
|  | CA | 591.4 | 975.0 | 7.8 | 3308.7 | 2915.9 |  | 0.220 | -0.650 | 0.2675 |
|  | FR | 175.5 | 340.9 | 1.9 | 2414.8 | 812.2 |  | 0.360 | -0.560 | 0.4996 |
|  | CO | 580.1 | 1440.7 | 1.3 | 17106.3 | 2704.6 |  | 0.420 | -0.520 | 0.2172 |
| ***EFAS*** | PA | 40.6 | 51.2 | 2.3 | 262.5 | 166.6 |  | 0.110 | -0.200 | 0.0948 |
|  | CH | 35.7 | 71.3 | 0.8 | 646.2 | 122.5 |  | 0.030 | -0.400 | 0.1398 |
|  | YU | 47.1 | 46.9 | 2.1 | 233.1 | 153.0 |  | -0.500 | -0.180 | 0.004 |
|  | CA | 59.5 | 74.0 | 1.7 | 403.0 | 169.4 |  | -0.100 | -0.840 | 0.1569 |
|  | FR | 50.1 | 60.1 | 2.1 | 451.3 | 180.0 |  | -0.090 | -0.740 | 0.5875 |
|  | CO | 39.3 | 46.8 | 1.1 | 243.0 | 154.7 |  | -0.240 | -0.970 | 0.379 |
| ***MCAV*** | PA | 153.6 | 185.4 | 7.4 | 1141.9 | 539.9 |  | -0.010 | -0.650 | 0.171 |
|  | CH | 203.3 | 305.1 | 7.4 | 1555.4 | 814.3 |  | 0.210 | -0.240 | 0.0599 |
|  | YU | 186.3 | 312.7 | 3.6 | 2248.4 | 625.8 |  | 0.160 | -0.420 | 0.5401 |
|  | CA | 254.0 | 528.1 | 5.1 | 4369.4 | 982.1 |  | 0.230 | -0.310 | 0.568 |
|  | FR | 234.2 | 292.2 | 11.0 | 1381.7 | 963.8 |  | 0.100 | -0.520 | 0.4157 |
|  | CO | 281.2 | 398.2 | 6.3 | 3176.3 | 997.0 |  | -0.020 | -0.400 | 0.3016 |
| ***MMEA*** | PA | 195.3 | 168.7 | 23.1 | 634.6 | 634.6 |  | -0.260 | -0.940 | 0.2233 |
|  | CH | 212.5 | 203.4 | 53.4 | 582.8 | 582.8 |  | 0.560 | -1.520 | 0.0033 |
|  | YU | 285.9 | 187.3 | 43.3 | 798.2 | 798.2 |  | -0.880 | 0.280 | 0.0014 |
|  | CA | 205.6 | 269.2 | 15.3 | 395.9 | 395.9 |  | -0.780 | 0.180 | 0.0001 |
|  | FR | 70.1 | 52.9 | 23.4 | 149.3 | 149.3 |  | 0.300 | -1.500 | 0.0001 |
|  | CO | 114.9 | 73.5 | 58.1 | 226.9 | 226.9 |  | 0.860 | -1.420 | 0.0008 |
| ***OANN*** | PA | 59.3 | 97.6 | 3.0 | 526.7 | 147.9 |  | 0.400 | 0.030 | 0.5663 |
|  | CH | 144.8 | 155.7 | 6.2 | 717.5 | 407.9 |  | -0.180 | -0.420 | 0.605 |
|  | YU | 209.9 | 681.0 | 3.5 | 3617.0 | 711.2 |  | 1.020 | 2.000 | 0.0015 |
|  | CA | 179.5 | 265.8 | 4.1 | 1854.7 | 654.6 |  | 0.040 | -0.250 | 0.0722 |
|  | FR | 169.9 | 303.2 | 2.0 | 3520.2 | 573.6 |  | 0.050 | 0.680 | 0.069 |
|  | CO | 194.1 | 562.7 | 2.2 | 11786.6 | 612.4 |  | 0.130 | 0.150 | 0.157 |
| ***PAST*** | PA | 37.3 | 31.4 | 2.2 | 247.5 | 103.3 |  | -0.120 | -0.420 | 0.0802 |
|  | CH | 73.8 | 64.8 | 5.5 | 400.8 | 186.1 |  | -0.190 | -0.190 | 0.1921 |
|  | YU | 74.1 | 63.3 | 4.0 | 603.3 | 180.2 |  | -0.450 | 0.360 | 0.0397 |
|  | CA | 75.1 | 81.1 | 5.3 | 737.4 | 181.8 |  | -0.010 | -0.310 | 0.096 |
|  | FR | 52.4 | 68.7 | 3.2 | 502.4 | 123.5 |  | 0.040 | 0.790 | 0.0094 |
|  | CO | 96.1 | 271.9 | 4.7 | 8478.4 | 236.6 |  | 0.080 | 0.580 | 0.0637 |
| ***PPOR*** | PA | 39.3 | 31.7 | 9.5 | 133.6 | 133.6 |  | 0.310 | -0.630 | 0.0561 |
|  | CH | 82.8 | 59.8 | 13.1 | 146.6 | 146.6 |  | -0.650 | -1.310 | 0.0001 |
|  | YU | 211.2 | 530.1 | 3.4 | 6901.7 | 861.1 |  | 0.420 | -0.040 | 0.1914 |
|  | CA | 93.6 | 154.6 | 3.3 | 913.9 | 432.1 |  | 0.490 | 0.020 | 0.0133 |
|  | FR | 168.4 | 656.5 | 1.8 | 13418.7 | 544.5 |  | 0.570 | 0.670 | 0.0206 |
|  | CO | 232.9 | 783.4 | 1.4 | 14378.0 | 758.8 |  | 0.430 | 0.260 | 0.0638 |
| ***PSTR*** | PA | 317.9 | 522.2 | 6.7 | 2135.0 | 1319.0 |  | 0.300 | -0.770 | 0.0251 |
|  | CH | 556.7 | 512.5 | 16.3 | 1185.7 | 1185.7 |  | -1.490 | -0.920 | 0.0001 |
|  | YU | 405.4 | 385.8 | 4.6 | 1048.0 | 1048.0 |  | -0.910 | -0.700 | 0.0187 |
|  | CA | 267.7 | 308.0 | 9.4 | 929.4 | 929.4 |  | -0.010 | -1.470 | 0.0788 |
|  | FR | 220.7 | 432.1 | 11.2 | 1737.4 | 1737.4 |  | 0.840 | -0.620 | 0.0723 |
|  | CO | 170.4 | 217.5 | 6.8 | 939.7 | 563.9 |  | -0.670 | -0.820 | 0.026 |
| ***SRAD*** | PA | 42.5 | 45.4 | 1.7 | 265.8 | 136.5 |  | -0.600 | -0.420 | 0.1581 |
|  | CH | 35.3 | 44.5 | 2.1 | 320.2 | 118.0 |  | 0.150 | -0.300 | 0.0739 |
|  | YU | 31.9 | 21.6 | 3.6 | 85.7 | 71.7 |  | -0.530 | -0.590 | 0.5073 |
|  | CA | 36.2 | 37.8 | 2.7 | 159.8 | 127.0 |  | -0.090 | -0.150 | 0.2228 |
|  | FR | 32.2 | 43.4 | 2.5 | 342.3 | 104.9 |  | 0.620 | 0.910 | 0.0197 |
|  | CO | 63.3 | 60.5 | 3.3 | 262.6 | 205.3 |  | -0.260 | -0.150 | 0.152 |
| ***SSID*** | PA | 85.9 | 144.9 | 0.6 | 1403.0 | 311.0 |  | 0.170 | 0.190 | 0.0669 |
|  | CH | 131.8 | 203.9 | 4.2 | 1572.6 | 489.8 |  | 0.230 | -0.450 | 0.6856 |
|  | YU | 133.0 | 216.2 | 3.3 | 1373.0 | 581.1 |  | 0.220 | -0.100 | 0.023 |
|  | CA | 150.1 | 217.4 | 2.4 | 1803.0 | 574.1 |  | 0.090 | -0.210 | 0.0849 |
|  | FR | 96.2 | 171.6 | 4.6 | 1342.1 | 391.2 |  | 0.740 | 0.380 | 0.0807 |
|  | CO | 178.2 | 253.2 | 3.1 | 1895.0 | 685.1 |  | -0.090 | 0.150 | 0.0835 |
